# Predictive value of serum adiponectin and hemoglobin levels for vascular cognitive impairment in ischemic stroke patients

**DOI:** 10.12669/pjms.38.3.5204

**Published:** 2022

**Authors:** Zhouling Li, Manlian Zhu, Cheng Meng, Hai Lin, Lifen Huang

**Affiliations:** 1 Zhouling Li, Department of Neurology, The Second People’s Hospital of Lishui, Lishui 323000, Zhejiang Province, China; 2 Manlian Zhu, Department of Neurology, The Second People’s Hospital of Lishui, Lishui 323000, Zhejiang Province, China; 3 Cheng Meng, Department of Neurology, The Second People’s Hospital of Lishui, Lishui 323000, Zhejiang Province, China; 4 Hai Lin, Department of Neurology, The Second People’s Hospital of Lishui, Lishui 323000, Zhejiang Province, China; 5 Lifen Huang, Department of Neurology, The Second People’s Hospital of Lishui, Lishui 323000, Zhejiang Province, China

**Keywords:** Adiponectin, Hemoglobin, Ischemic stroke, Vascular cognitive impairment

## Abstract

**Objectives:**

To investigate the potential predictive value of serum adiponectin (APN) and hemoglobin (Hb) levels for the occurrence of vascular cognitive impairment in ischemic stroke patients.

**Methods:**

Eighty ischemic stroke patients, admitted to our hospital between June 2019 and November 2020, were retrospectively divided into no cognitive impairment (NCI) group (n=43) and cognitive impairment (CI) group (n=37) based on Montreal Cognitive Assessment (MoCA) scale scoring at three months follow-up. ELISA was used to assess serum Hb and APN levels and receiver operating characteristic (ROC) curves were created to evaluate correlation.

**Results:**

Serum APN and Hb levels were lower in the vascular cognitive impairment group compared to non-impaired counterparts. Pearson correlation analysis showed that both APN and Hb levels were positively correlated with MoCA scores. Area under curve analysis indicated predictive value for serum APN and Hb for predicting cognitive impairment in ischemic stroke patients.

**Conclusion:**

Serum APN and Hb levels in ischemic stroke patients have value for predicting vascular cognitive impairment and may be suitable for helping dictate treatment planning.

## INTRODUCTION

Ischemic stroke incidence is high in the Chinese elderly population, as is mortality and disability rate. Current evidence suggests that 25–30% of ischaemic stroke patients develop vascular cognitive impairment or vascular dementia.^1^,^2^ Early diagnosis and intervention can effectively delay or prevent vascular cognitive impairment from developing into vascular dementia.^3^,^4^ Vascular dementia pathogenesis involves oxidative stress responses, which can be detected by measuring serum hemoglobin (Hb) levels.^5^ Moreover, adiponectin (APN) has been recently found to improve vascular dementia in patients with atherosclerosis, glucose metabolism disorder, and hypertension.^6^ However, the value of serum APN and Hb levels for predicting vascular cognitive impairment in ischemic stroke patients has not been investigated. This study examined the serum APN and Hb levels of 80 ischemic stroke patients and used this data to examine the relationship between these parameters and vascular cognitive impairment probability.

## METHODS

For this retrospective study, medical records of ischemic stroke patients hospitalized between June 2019 and November 2020 in the Second People’s Hospital of Li Shui were evaluated, and 80 patients were included in the study. Study protocol was approved by the ethics committee of the Second People’s Hospital of Li Shui (No. 2021012).

### Inclusion criteria:

- Stroke onset time within 24 hours.
- Ischemic stroke was diagnosed in accordance with the 2014 Chinese National Guidelines for the Diagnosis of Acute Ischemic Stroke.^7^
- The patient had no prodromal symptoms before onset, and the cognitive function, motor function and social adaptability were at a normal level.

### Exclusion criteria:

- Patients with a modified Rankin score^8^ under 5 points.
- Patients with other severe medical diseases such as liver/kidney failure and acute myocardial infarction.
- Patients with hearing impairment, visual impairment, dysarthria, aphasia, or other symptoms preventing the independent completion of the cognitive function evaluation.
- Patients with Hamilton Depression Scale^9^ scores equal or greater than 8.
- Brain tumor patients.
- Patients with connective tissue, rheumatic, immune, or infectious diseases.
- Patient with cognitive impairment prior to ischemic stroke.

### Additionally, exclusion criteria during the follow-up period included:

- Patients diagnosed with serious diseases (such as myocardial infarction or severe trauma) during the follow-up period.
- Patients who were unwilling to continue the study or who were withdrawn by their families.
- Patients who suffered another ischemic stroke.
- Patients who died or were not followed-up.

### Detection of Serum APN and Hemoglobin

For all hospitalized ischemic stroke patients, fasting venous blood (3-4 ml) was collected on the morning of the day after admission. Collected blood was centrifuged at 3000 rpm for 10 minutes to separate and collect upper serum, which was stored at -20°C. Serum levels of Hb and APN were detected using immunofluorescence assay (ELISA) according to manufacturer’s instructions.

### Patient Follow-Up

All patients, included in the study, had records of the follow-up assessment three months after treatment, where their cognitive function was evaluated using the Montreal Cognitive Assessment Scale (MoCA).^10^ by two neurologists. Based on the recorded data, patients with MOCA scores greater or equal to 23 (of a maximum of 30) were retrospectively assigned to the no cognitive impairment (NCI) group (n = 43), while those with scores under 23 were assigned to the cognitive impairment (CI) group (n = 37).

### Observation Indices were as follows

basic patient data, collected during hospitalization, including age, gender, chronic diseases (atrial fibrillation, chronic obstructive pulmonary disease, hyperlipidemia, and coronary heart disease), smoking, drinking, and education level. Homocysteine (Hcy), high-sensitivity C-reactive protein (hs CRP), and systolic blood pressures (SBP) were also recorded. Hyperlipidemia was defined as low density lipoprotein (LDL) ≥ 3.37 mmol/l, triglyceride levels ≥ 1.70 mmol/l, or serum total cholesterol ≥ 5.18 mmol/l. Drinking was defined as consuming alcohol at least once a week over a period of six months. Smoking was defined as one cigarette per day over a period of six months. Homocysteine levels ≥ 15 mmol/l were considered as elevated, with blood hypersensitive C-reactive protein levels > 3 mg/l considered elevated.

### Data Analysis

All data were analyzed by SPSS 20.0 software. For data expressed as mean ± SD, t-tests were used to examine relationships between groups. Chi square tests were used when data was expressed as percentages. Pearson correlation analysis was used to analyze correlations between serum APN, Hb, and MOCA scores. Receiver operating characteristic curves (ROC) were used to evaluate the diagnostic ability of APN and Hb for vascular cognitive impairment after ischemic stroke. Logistic regression analysis was used to analyze influencing factors for vascular cognitive impairment in ischemic stroke. P < 0.05 indicated statistical significance.

## RESULTS

Serum APN and Hb levels in patients in the CI group were lower than those without vascular cognitive impairment (NCI group, P < 0.05, Table-I). Pearson correlation analysis showed that serum APN and Hb levels were positively correlated with MOCA score (r = 0.447 and 0.452, respectively, P < 0.01).

**Table I:** Serum APN and Hb levels in patients with and without vascular cognitive impairment.

Group	n	APN(mg/L)	HB(g/L)
CI group	37	5.38±2.74	102.53±12.36
NCI group	43	9.39±2.41	136.48±10.38
T		6.96	13.35
P		0.00	0.00

NCI, no cognitive impairment; CI, cognitive impairment.

**Fig.1:**
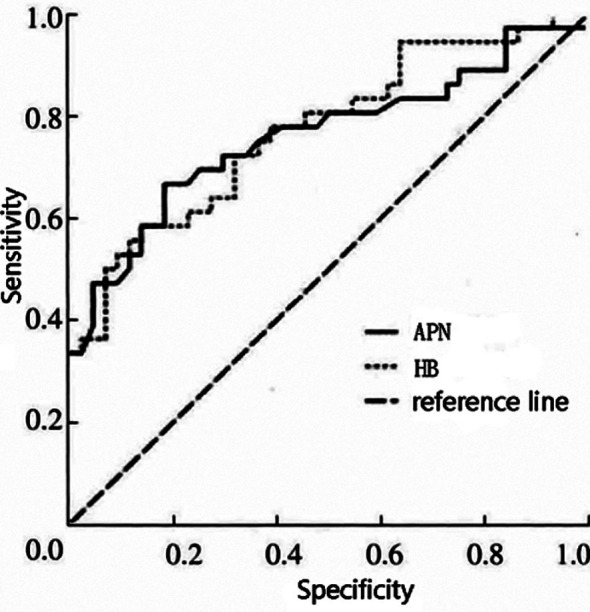
ROC curve analysis.

The area under the curve (AUC) for the serum APN and Hb ROC curves for the diagnosis of vascular cognitive impairment in patients with ischemic stroke were 0.768 (95% Cl: 0.652-0.881) and 0.782 (96% Cl: 0.672-0.884), respectively (Fig.1). This showed positive significance for predicting vascular cognitive impairment in patients with ischemic stroke (P < 0.05).

Univariate analysis showed that serum VPN, Hb, Hcy, hs CRP, and SBP were associated with vascular cognitive impairment in patients with ischemic stroke (P < 0.05). Atrial fibrillation, chronic obstructive pulmonary disease, hyperlipidemia, coronary heart disease, smoking, drinking, educational level, age, and gender were not associated (P > 0.05, Table-II). Logistic regression analysis showed that serum APN, Hb levels, and homocysteine were independent risk factors for vascular cognitive impairment in patients with ischemic stroke (P < 0.05, Table-III).

**Table II:** Single factor analysis.

Group		CI group	NCI group	t/χ^2^	P
Number of cases		37	43		
Age		65.38±8.93	64.92±9.31	0.22	0.823
Gender	Male	19	21	0.05	
	Female	18	22		
Education level	Primary school and below	11	13	0.028	
	Junior high school and senior high school	17	19		
	Junior college or above	9	11		
Drink wine	Yes	12	14	0	
	No	25	29		
Smoke	Yes	13	16	0.037	
	No	24	27		
Systolic blood pressure ≥ 140 mmHg	Yes	23	14	7.011	
	No	14	29		
Coronary heart disease	Yes	12	17	0.434	
	No	25	26		
Hyperlipidemia	Yes	18	20	0.036	
	No	19	23		
Chronic obstructive pulmonary disease	Yes	7	10	0.224	
	No	30	33		
Atrial fibrillation	Yes	9	13	0.348	
	No	28	30		
High sensitivity C-reactive protein increased	Yes	28	20	7.048	
	No	9	23		
Elevated homocysteine	Yes	27	9	21.763	
	No	10	34		
APN(mg/L)		5.38±2.74	9.39±2.41	6.96	
HB(g/L)		102.53±12.36	136.48±10.38	13.35	

NCI, no cognitive impairment; CI, cognitive impairment; APN, adiponectin; Hb, hemoglobin.

**Table III:** Multivariate analysis.

Item	Β	S.E.	WaldX²	P	OR	95%CI	
APN	-0.714	0.264	7.315	0.007	0.490	0.292	0.822
HB	-0.813	0.231	12.387	0.000	0.444	0.282	0.698
Systolic pressure	0.783	0.475	2.717	0.099	2.188	0.862	5.551
High sensitivity C-reactive protein	0.989	0.518	3.645	0.056	2.689	0.974	7.421
Homocysteine	1.465	0.593	6.103	0.013	4.328	1.354	13.836

APN, adiponectin; Hb, hemoglobin.

## DISCUSSION

This study showed that serum APN and Hb levels in ischemic stroke patients with vascular cognitive impairment were lower than patients with no cognitive impairment. Recent epidemiological studies have suggested an increase in the incidence of ischemic stroke in China, which leads to an increased prevalence of varying degrees of vascular cognitive impairment and dementia.^11^–^13^ Clinically, motor dysfunction is the primary concern when treating ischemic stroke patients. However, more data is showing that improving cognitive function is very important for functional recovery and quality of life.^14^,^15^ APN is a very active adipocyte secretory factor that combines with receptors AdipoR1 and R2 to exert its biological functions.^16^–^18^ Previous studies that have found that neurodegenerative diseases, cardiovascular diseases, hypertension, diabetes, abnormal lipid metabolism, obesity, and other diseases are closely related to APN levels.^19^–^21^. APN and its receptors are abundantly expressed in the brain and may have a crucial effect on cognitive function. However, there is still no consensus on the correlation between APN levels and cognitive function. Studies by Kamogawa et al. and Une et al. showed that increased plasma APN levels were associated with better cognitive function in males, but not in females. Roberts et al. found no significant difference in circulating APN levels was visible between mild cognitive impairment patients and controls. In contrast, Teixeira et al. reported that in patients with mild CI and Alzheimer’s disease, low circulating APN levels were associated with cognitive dysfunction.^22^ Their conclusions are in agreement with the results of our study that in ischemic stroke patients low APN levels correlate with lower cognitive function.

Anemia and overall decreased Hb levels reported in up to 40% of patients with acute ischemic stroke.^23^–^25^ Studies indicate that decreased Hb levels lead to increased oxidative stress in neuronal cells and subsequent cognitive dysfunction.^26^ A current systemic review and meta-analysis found a direct association between anemia and cognitive function deterioration and dementia in older patients > 65 years old.^27^,^28^ In addition to elevated oxidative stress, decreased Hb levels also promotes hypoxia by lowering blood oxygen capacity.^29^,^30^ Anemia can also induce oxidative stress through endothelial damage.^31^,^32^ In our study, decrease in Hb level was strongly associated with the decrease in cognitive function of patients in the CI group as compared to patients without cognitive impairment. Pearson correlation analysis in our study showed that in ischemic stroke patients, the serum APN and Hb levels were positively correlated with MOCA scores, indicating a linear relationship between serum APN and Hb levels and vascular cognitive impairment severity. Finally, ROC analysis showed that both serum APN and Hb levels had a better predictive function for predicting vascular cognitive impairment in patients with ischemic stroke, but that the AUC for serum Hb was larger than that for serum APN, indicating that serum Hb levels are a better predictive indicator than serum APN levels.

### Limitations of the study

This study has several limitations. There was no long-term follow-up information, nor was there any dynamic observation of serum APN and Hb levels over time. As such, we believe that these findings need to be further confirmed via a long-term longitudinal study with a larger sample size.

## CONCLUSIONS

The data presented here indicates that serum APN and Hb levels correlate with the development and severity of vascular cognitive impairment in ischemic stroke patients. Therefore, these values may be predictive for vascular cognitive impairment, and can be used for optimizing interventional strategies.

### Authors’ contributions:

**ZL:** Conceived and designed the study and is responsible for integrity of the study.

**MZ, CM & HL:** Collected the data and performed the analysis.

**ZL:** Was involved in the writing of the manuscript.

**LH:** Data analysis, interpretation, Edited the manuscript.

All authors have read and approved the final manuscript.

## References

[1] Dula AN, Mlynash M, Zuck ND, Albers GW, Warach SJ (2020). DEFUSE 3 Investigators. Neuroimaging in Ischemic Stroke Is Different Between Men and Women in the DEFUSE 3 Cohort. Stroke.

[2] Kalaria RN, Akinyemi R, Ihara M (2016). Stroke injury, cognitive impairment and vascular dementia. Biochim Biophys Acta.

[3] Christ N, Mocke V, Fluri F (2019). Cerebral microbleeds are associated with cognitive decline early after ischemic stroke. J Neurol.

[4] Seetlani NK, Kumar N, Imran K, Ali A, Shams N, Sheikh T (2016). Alzheimer and vascular dementia in the elderly patients. Pak J Med Sci.

[5] Muller CR, Williams AT, Munoz CJ, Eaker AM, Breton AN, Palmer AF (2021). Safety profile of high molecular weight polymerized hemoglobins. Transfusion.

[6] Lin Y-H, Jiang T-X, Hu S-X, Shi Y-H (2020). Association between serum adiponectin concentrations and chronic obstructive pulmonary disease:a meta-analysis. Biosci Rep.

[7] Chinese Neurology Association. Stroke Committee. Guideline of diagnosis and treatment in acute ischemic stroke 2014 (2015). Chinese J Neurol.

[8] Rankin J (1957). Cerebral vascular accidents in patients over the age of 60 II. Prognosis. Scott Med J.

[9] Hamilton M (1960). A rating scale for depression. J Neurol Neurosurg Psychiatry.

[10] Nasreddine ZS, Phillips NA, Bédirian V, Charbonneau S, Whitehead V, Collin I (2005). The Montreal Cognitive Assessment, MoCA:A Brief Screening Tool for Mild Cognitive Impairment. J Am Geriatrics Soc.

[11] Liu M, Wu B, Wang W-Z, Lee L-M, Zhang S-H, Kong L-Z (2007). Stroke in China:epidemiology, prevention, and management strategies. Lancet Neurol.

[12] Shao SJ, Zhang GZ, Zhao L, Huo FR, Ma HB, Zhu L (2020). Microcatheter infusion of bolus-dose tirofiban for acute ischemic stroke due to distal intracranial artery occlusion. Medicine (Baltimore).

[13] Wang W, Liu C, Ying Z, Lei X, Wang C, Huo J (2019). Particulate air pollution and ischemic stroke hospitalization:How the associations vary by constituents in Shanghai, China. Sci Total Environ.

[14] Bejot Y, Duloquin G, Crespy V, Durier J, Garnier L, Graber M (2020). Influence of Preexisting Cognitive Impairment on Clinical Severity of Ischemic Stroke. Stroke.

[15] Zheng L, Yu M, Lin R, Wang Y, Zhuo Z, Cheng N (2020). Rhythmic light flicker rescues hippocampal low gamma and protects ischemic neurons by enhancing presynaptic plasticity. Nat Commun.

[16] Yadav R, Aggarwal S, Singh A, Mir R (2019). Effect of surgically induced weight loss on serum adiponectin levels and its association with the gene expression in visceral adipose tissue of morbidly obese individuals. Clin Chimica Acta.

[17] Liang S, Li H, Shen X, Liu R (2019). Increased serum adiponectin predicts improved coronary flow and clinical outcomes in patients with ST-segment elevation myocardial infarction treated by primary percutaneous coronary intervention. J Clin Lab Anal.

[18] Munoz-Prieto A, Martínez-Subiela S, Cerón JJ, Tvarijonaviciute A (2019). A new highly sensitive immunoassay for the detection of adiponectin in serum and saliva of dogs and its application in obesity and canine leishmaniosis. Res Vet Sci.

[19] Sanz B, Arrieta H, Hervás G, Rezpla-Pardo C, Ruiz-Litage F, Iturburu M (2019). Serum adiponectin is associated with body composition and cognitive and psychological status in older adults living in long-term nursing homes. Exp Gerontol.

[20] Yu Z, Tang S, Ma H, Duan H, Zeng Y (2019). Association of serum adiponectin with breast cancer. Medicine (Baltimore).

[21] Liu W, Zhou X, Li Y, Zhang S, Cai X, Zhuang R (2020). Serum leptin, resistin, and adiponectin levels in obese and non-obese patients with newly diagnosed type 2 diabetes mellitus. Medicine (Baltimore).

[22] Teixeira AL, Diniz BS, Campos AC, Miranda AS, Rocha NP, Talib LL (2013). Decreased levels of circulating adiponectin in mild cognitive impairment and Alzheimer's disease. Neuromolecular Med.

[23] Tanne D, Molshatzki N, Merzeliak O, Tsabari R, Toashi M, Schwammenthal Y (2010). Anemia status, hemoglobin concentration and outcome after acute stroke:a cohort study. BMC Neurol.

[24] Corona LP, Duarte YA, de O, Lebrão ML (2014). Prevalence of anemia and associated factors in older adults:evidence from the SABE Study. Rev Saude Publica.

[25] Li L, Yiin GS, Geraghty OC, Schulz UG, Kuker W, Mehta W (2015). Incidence, outcome, risk factors, and long-term prognosis of cryptogenic transient ischaemic attack and ischaemic stroke:a population-based study. Lancet Neurol.

[26] Nathan DM (1981). Labile glycosylated hemoglobin contributes to hemoglobin A1 as measured by liquid chromatography or electrophoresis. Clin Chem.

[27] Andro M, Le Squere P, Estivin S, Gentric A (2013). Anaemia and cognitive performances in the elderly:a systematic review. Eur J Neurol.

[28] Shah RC, Buchman AS, Wilson RS, Leurgans SE, Bennett DA (2011). Hemoglobin level in older persons and incident Alzheimer disease:prospective cohort analysis. Neurology.

[29] Liu X, Li J, Chen J, Rohimah S, Tian H, Wang J (2020). Fano resonance based on D-shaped waveguide structure and its application for human hemoglobin detection. Appl Opt.

[30] Jordan LC, Rodeghier M, Donahue MJ, DeBaun MR (2020). Reduction in TCD velocity after regular blood transfusion therapy is associated with a change in hemoglobin S fraction in sickle cell anemia. Am J Hematol.

[31] Ran R, Zhang R, Xie Y, Yin Z (2021). Decreased hemoglobin as a quantifiable indicator of renal arterial embolization in post-percutaneous nephrolithotomy hemorrhage. Urolithiasis.

[32] Nugent WH, Carr DA, Macko AR, Song BK (2020). Physiological and microvascular responses to hemoglobin concentration-targeted hemolytic anemia in rats. Journal of Applied Physiology.

